# Early pregnancy serum levels of perfluoroalkyl substances and risk of preeclampsia in Swedish women

**DOI:** 10.1038/s41598-019-45483-7

**Published:** 2019-06-24

**Authors:** Sverre Wikström, Christian H. Lindh, Huan Shu, Carl-Gustaf Bornehag

**Affiliations:** 1 School of Medical Sciences, Örebro University, Örebro, Sweden; 2 Department of Health Sciences, Karlstad University, Karlstad, Sweden; 3 Division of Occupational and Environmental Medicine, Lund University, Lund, Sweden; 4 Department of Environmental Science and Analytical Chemistry, Stockholm University, Stockholm, Sweden; 5 Department of Preventive Medicine, Icahn School of Medicine at Mount Sinai, New York, USA

**Keywords:** Risk factors, Epidemiology

## Abstract

Preeclampsia is a major cause of maternal and fetal morbidity. Emerging research shows an association with environmental exposures. The present aim was to investigate associations between early pregnancy serum levels of perfluoroalkyl substances (PFAS) and preeclampsia. Within the Swedish SELMA study, eight PFAS were measured at median 10 gestational weeks and cases of preeclampsia were postnatally identified from registers. Associations between individual PFAS and preeclampsia were assessed, adjusting for parity, age, weight and smoking. Out of 1,773 women in the study group, 64 (3.6%), developed preeclampsia. A doubling of PFOS and PFNA exposure, corresponding to an inter-quartile increase, was associated with an increased risk for preeclampsia of about 38–53% respectively. Serum PFOS within the highest quartile was associated with an odds ratio of 2.68 (CI 95%: 1.17–6.12), equal to the increased risk associated with nulliparity, when compared to exposure in the first quartile. The same associations were identified, although with higher risk estimates, in analyses restricted to nulliparous women. For other PFAS, there were no associations. In conclusion and consistent with limited previous research only on PFOS, increasing serum levels of PFOS and PFNA during early pregnancy were associated with a clinically relevant risk of preeclampsia, adjusting for established confounders.

## Introduction

Preeclampsia is a condition during pregnancy defined by hypertension and proteinuria. It affects around 2–8% of pregnancies^1^ and is a major cause of maternal and fetal morbidity, and worldwide responsible for more than 10% of maternal mortality^1,2^. Consequences to the fetus include increased mortality rates, intrauterine growth restriction, preterm birth and developmental impairments^1^. The mechanisms behind preeclampsia are still not understood although multiple etiologies are proposed. These include abnormal placental implantation, genetic disposition, oxidative stress, metabolic as well as vascular disturbances and inflammatory mechanisms^3,4^. Major clinical risk factors are nulliparity, previous history of preeclampsia, heredity, higher maternal age, chronic hypertension or renal disease, diabetes, multiple gestation and overweight^3,5^. In addition, also environmental factors have been suggested to contribute to preeclampsia^6,7^.

Perfluoroalkyl substances (PFAS) are organic compounds used for many decades to make everyday products more resistant to stains, grease, and water. Products containing PFAS range from non-stick frying pans, waterproof clothing, stain resistant fabrics to food packaging, and cosmetics^8–10^. More recently it has been identified in fire-fighting foam, and by such use contaminating drinking water sources giving rise to high serum levels in humans^11^. Research has revealed that several PFAS are very persistent and have the potential to bioaccumulation in blood and liver^12^. PFAS have not only been detected in the environment (soil and water) and in animals, but also in human blood samples throughout the world^13–15^.

Over the past decade, new evidence has implicated an association between exposure for PFAS and human health and development. Johnson, *et al*.^16^ included 19 datasets from nine countries in a systematic review analysis and concluded that there is “sufficient” human evidence that prenatal exposure to perfluorooctanoic acid (PFOA) reduce foetal growth, in line also with rodent experimental studies^17^. However, a more recent meta-analysis by Steenland *et al*.^18^ raise concerns about confounding or reverse causality in the association between PFAS and birth weight, especially in studies utilizing blood sampling late in pregnancy. Some studies have also linked PFAS exposure to decreased fertility, although with inconclusive findings^19,20^, as well as changes to the immune system^21^.

Despite this, and the emerging evidence that preeclampsia risk may be influenced by environmental exposures such as air pollution and persistent organic pollutants^6,7^, there are few investigations of PFAS exposure as potential risk factor of preeclampsia^22–24^. In studies of high exposed American populations, indirect estimates of exposures to PFOA and perfluorooctane sulfonate (PFOS) were associated with maternally reported preeclampsia^22,23^, but no other PFAS were studied. In addition, a recent Norwegian study reported weak associations between preeclampsia and mid pregnancy serum concentrations of perfluoroheptane sulfonate (PFHpS) and PFOS^24^. In order to limit the potential of confounding (e.g from pregnancy related changes in PFAS serum concentrations), it is however important to study preeclampsia risk in relation to directly measured PFAS exposures early in pregnancy.

Consequently, the present study was set up in order to investigate associations between early prenatal (median 10 gestational weeks) serum levels of eight individual PFAS and preeclampsia during the same pregnancy in the Swedish Environmental Longitudinal, Mother and child, Asthma and allergy (SELMA) pregnancy cohort.

## Results

Outcome data and all co-variate data from the Medical Birth Register, together with exposure data were available for 1,773 women, constituting the present study group. Within this we identified 64 cases of preeclampsia (of which 42 were nulliparous), corresponding to a prevalence of 3.6% (Table 1). There were no case of eclampsia or HELLP-syndrome (i.e., Hemolysis, Elevated Liver enzymes, Low platelet count Syndrome), and none had pre-pregnancy hypertension. Women with preeclampsia were significantly more often nulliparous (unadjusted OR for preeclampsia was 2.2 [95% CI: 1.3–3.7]), had higher body weight, and gave birth to infants with lower birth weight, as compared to controls (Table 1).

**Table 1:** Description of the study population of 1,773 women.

	Preeclampsia N = 64	Controls N = 1,709	p-value^a^
Nulliparous (n)	42 (65.6%)	770 (45.1%)	0.002
Maternal age (year)	32 (28–35)	31 (28–34)	0.324
Maternal weight (kg)	71 (64–84)	67 (60–76)	0.009
Smoke exposed (n)	7 (10.9%)	221 (12.9%)	0.616
Female fetus (n)	31 (48%)	810 (47%)	0.965
Multiple gestation (n)	1 (1.5%)	15(0.9%)	0.698
Birth weight (g)	3,322 (2,715–3,810)	3,640 (3,282–4,000)	<0.001

Values are n (%) or median (IQR) for continuous variables.

^a^P-values for differences between preeclampsia cases and the remaining women (controls) tested by Chi^2^, Fisher’s Exact or Mann-Whitney U test as appropriate.

Among the eight studied compounds, PFOS had the highest concentrations, followed by PFOA. The distribution of respectively PFAS levels in mothers’ serum at median 10 gestational weeks is presented in Table 2. Due to the large number (>50%) of samples with levels below the Limit of Detection (LOD), we performed no analyses regarding PFDoDA.

**Table 2:** Serum concentrations (ng/mL) of eight PFAS at median week 10 of pregnancy for 1,773 women.

Compound	Geometric mean [95% CI]	Median (IQR)	95^th^ %	LOD^a^	Above LOD (%)
PFOS	5.34 [5.21–5.48]	5.39 (3.95–7.61)	12.41	0.06	100
PFOA	1.61 [1.57–1.66]	1.61 (1.12–2.31)	4.10	0.02	100
PFHxS	1.32 [1.28–1.36]	1.23 (0.87–1.97)	3.66	0.03	100
PFNA	0.54 [0.53–0.56]	0.53 (0.39–0.74)	1.32	0.01	100
PFDA	0.26 [0.26–0.27]	0.26 (0.19–0.35)	0.61	0.02	100
PFUnDA	0.21 [0.21–0.22]	0.23 (0.15–0.33)	0.54	0.02	99.5
PFHpA	0.018 [0.017–0.019]	0.02 (<LOD-0.04)	0.097	0.01	73.8
PFDoDA^2^	0.027 [0.026–0.027]	<LOD (<LOD-0.05)	0.08	0.03	46.6

PFDoDA was excluded from further analyses due to the large number of samples below LOD.

^a^LOD = Limit of Detection.

### Prenatal PFAS serum concentrations and preeclampsia

In crude analyzes there were significant associations between preeclampsia and serum levels of PFOS, PFOA and PFNA (Table 3**)**. With adjustments for parity, age, body weight and smoke exposure, significant associations remained for PFOS and PFNA. When the same adjusted regression models were restricted to nulliparous women, OR estimates for preeclampsia were higher for both PFOS and PFNA. Exclusion of twin pregnancies or inclusion of education level and calendar month of sampling in the regression models did not change Odds Ratio estimates (data not shown).

**Table 3:** Associations between serum levels of seven PFAS (log base 2) and preeclampsia in (a) the full study group (N = 1,773 women and n = 64 cases) and (b) restricted to nulliparous women (N = 812 and n = 42 cases).

Compound	Crude OR (95% CI)	Adjusted^a^ OR (95% CI)
**(a) All women (n = 64 cases of preeclampsia)**		
PFOS	1.74 [1.23–2.46]	1.53 [1.07–2.20]
PFOA	1.53 [1.13–2.07]	1.31 [0.93–1.87]
PFHxS	1.21 [0.91–1.62]	1.16 [0.86–1.56]
PFNA	1.50 [1.11–2.01]	1.38 [1.01–1.89]
PFDA	1.19 [0.85–1.66]	1.11 [0.79–1.59]
PFUnDA	0.93 [0.72–1.21]	0.89 [0.68–1.17]
PFHpA	1.04 [0.88–1.23]	1.02 [0.86–1.21]
**(b) Nulliparous women (n = 42 cases of preeclampsia)**		
PFOS	2.06 [1.29–3.39]	2.02 [1.26–3.29]
PFOA	1.36 [0.89–2.17]	1.38 [0.90–2.21]
PFHxS	1.23 [0.84–1.78]	1.24 [0.85–1.82]
PFNA	1.44 [1.01–2.06]	1.50 [1.04–2.16]
PFDA	1.24 [0.83–1.84]	1.29 [0.86–1.96]
PFUnDA	1.00 [0.72–1.41]	0.99 [0.69–1.42]
PFHpA	0.99 [0.81–1.21]	1.00 [0.82–1.22]

Associations expressed as crude and adjusted odds ratios.

^a^Adjustments made for parity (Table *a* only), and the women’s age, body weight, and smoke exposure.

**Figure 1:**
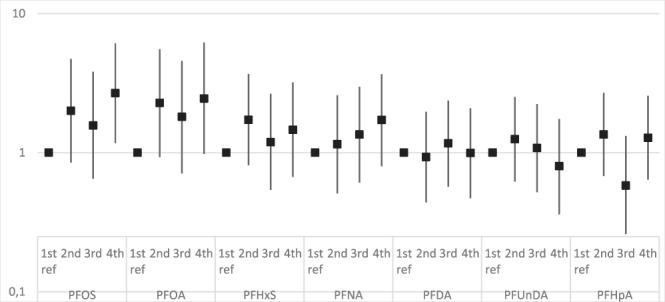
Odds ratios [95% CI] for preeclampsia per quartiles of respectively PFAS serum level, with first quartile exposure as reference. Adjusted for women’s parity, age, body weight and smoke exposure.

As analyzed per quartiles of PFAS exposures with the same co-variates as above, there was significantly increased risk for preeclampsia in the highest quartile range of PFOS exposure when compared with first quartile (OR = 2.68 [95% CI: 1.17–6.12]), however, for PFOA and PFNA, the increased risk for the highest quartile didn´t become significant (OR = 2.40 [95% CI: 0.95–6.06], and OR = 1.72 [95% CI: 0.80–3.67], respectively. Fig. 1).

## Discussion

In this analysis within a prospective pregnancy cohort study we show that increasing levels of serum PFOS and PFNA at median 10 weeks of gestation were associated with preeclampsia during the same pregnancy when adjusted for potential confounders. For PFOA, there were indications of a similar association according to crude analyses. In adjusted analyses however, the association between PFOA and preeclampsia did not reach statistical significance, which may be a matter of statistical power. For the other four assessed PFASs no significant results were found. Importantly, the associations between PFAS and preeclampsia were highly evident also in nulliparous women, where the highest OR estimates of our study was identified.

The association between PFAS exposure and preeclampsia has been indicated in other studies^22–24^. In studies of a large U.S. sample, (known as the “*C8 Health Project*”), model estimates of historical PFOA exposure based on residential history, was assessed by Savitz *et al*., in relation to self-reported preeclampsia^22^. An increase from the 25^th^ to the 75^th^ percentile in PFOA exposure was associated with an OR of 1.13[95% CI: 1.0–1.6] for preeclampsia^22^. In the same area, Stein *et al*., investigated self-reported preeclampsia in pregnancies during the five years prior to serum measurements of PFOA and PFOS. The authors report an OR of 1.3[95% CI: 0.9–1.9] for preeclampsia in women with PFOA levels above as compared to below the mean, and similar an OR of 1.3[95% CI: 1.1–1.7] for PFOS levels above vs below mean^23^. In the large Norwegian *MoBa* cohort, nulliparous women had nine PFAS measured in mid-pregnancy. Increased levels of PFOS and PFHpS had minor associations with preeclampsia risk (Hazard Ratio = 1.13[95% CI: 0.84–1.52] per ln-unit increase in PFOS)^24^, while PFNA exposure showed no association with preeclampsia.

Consequently there is previous support, although limited, for an association between PFOS and PFOA exposure (but not PFNA) and preeclampsia. There are however important differences between the few conducted studies. While the Norwegian *MoBa* study, like our, was based on Medical Birth Registry reports by health care professionals, the authors from the *C8 Health Project* (investigating questionnaire self-reports of preeclampsia), express concerns about diagnostic misclassification. This since preeclampsia incidence was unexpectedly high (7%) in that cohort. The prevalence of preeclampsia in the present study (3.6%) however, is the expected level in a Swedish population^25^, very similar to U.S or Norwegian prevalence data^26,27^.

Exposure measures also differ between studies, with indirect measures (PFAS model estimates or inclusion of pregnancies five years preceding serum measurements in the *C8 Health Project* reports), mid-pregnancy serum measurements (*MoBa*), and early pregnancy serum measurements (the present SELMA study), respectively. The most evident difference between the present study and the previous is consequently the differences in exposure measures and levels. Median PFOS and PFOA in all previous studies were considerably higher than in our cohort and studies in populations with different ranges of community exposure are important. PFOS concentrations were in median 13.6 ng/mL^23^ and 12.9 ng/mL^24^, as compared with 5.4 ng/mL in the present cohort. In the most comparable previous study^24^, mid pregnancy PFOS levels of the 5:th percentile was well above the 50^th^ percentile of our samples. For PFOA, median levels of the *C8 Health Project* were 15.9 and 48.8 ng/mL and in the Norwegian *MoBa* sample 2.78 ng/mL^22–24^, as compared to 1.6 ng/mL in our study. For other PFAS of specific interest for comparison, *MoBa* serum concentrations of PFNA were very similar to ours. Detailed data on the distribution of PFOS, PFOA and PFNA concentrations in the present study is provided in Supplementary Information (Figs 2–4).

Other environmental exposures have previously been found associated with increased risk of preeclampsia: A meta-analysis of 17 studies indicate that ambient air pollution increases the risk of pregnancy-induced hypertensive disorders such as preeclampsia^7^ and studies have shown specifically an association between air-pollutant exposure and preeclampsia risk^28^.

Mechanisms behind an association between PFAS exposure and preeclampsia is yet to be resolved but the concerns about immunologic effects from PFAS may be considered. The vascular disturbances that play a key role in preeclampsia development, include endothelial dysfunction associated also with oxidative stress and increased inflammatory response^3,4^. Given that PFOS have been shown to induce oxidative stress in human endothelial cells^29^, and the support for immune system changes to PFOS exposure^21,30^, one can speculate in pathways involving oxidative stress and inflammation behind the present associations. This may as well be a mechanism in common, explaining the increased preeclampsia risk from exposure both to PFAS and to air pollutions, as supported by the extensive literature on pathophysiological disturbances from air pollution^31^.

The present study has its strengths in the quality of both exposure and outcome data. We consider the Swedish Birth Register data highly valid, constituting post-partal reports also including diagnostic information from antenatal care, and preeclampsia prevalence was in agreement with previous data. Analyses were adjusted for established major risk factors. The importance of these risk factors (importantly including nulliparity) was very similar to what is previously known^3,5^, supporting a high quality of our data.

There are however still (minor) possibilities of residual confounding. Information on previous history of preeclampsia and heredity was not available in the present study (however unlikely to be associated with early pregnancy PFAS levels). Among other clinical risk factors for preeclampsia is multiple birth, but exclusion of twin pregnancies did not change the results and twin pregnancy was not associated with PFAS serum levels (data not shown). Pre-pregnancy renal dysfunction is a well-known risk factor for preeclampsia, but may also directly affect serum PFAS levels through impaired renal clearance, with potential of confounding the association between PFAS and preeclampsia. However, in the present study no woman with preeclampsia suffered from pre-pregnancy essential hypertension or known pre-pregnancy renal dysfunction, and only one woman with preeclampsia had pre-pregnancy diabetes. Still we can’t rule out that the present findings may be affected to a minor degree by pre-pregnancy glomerular filtration rate (GFR). Also in absence of preeclampsia, sampling during late pregnancy is related to confounding due to GFR-changes, plasma expansion and serum albumin changes during pregnancy^32^. The present serum samples were drawn at median 10 weeks of gestation, well before development of preeclampsia or pregnancy related changes in PFAS levels^32^. We therefore find it very unlikely that developing preeclampsia in itself affected the serum concentrations in any direction and we regard the early sampling an important strength of this study. Additional and unmeasured environmental exposures may influence the risk of preeclampsia but we find no reason to believe that any such exposure would also correlate with PFAS exposures. Here we performed statistical analyses compound by compound without correction for multiple comparisons since the analyses were hypothesis driven based on previous indications. We however find it important for the future that exposures to several PFAS may be inter-correlated due to common sources and correlations between PFAS levels in the present study are therefore presented in Supplementary Information (Table 4). We notice that preeclampsia Odds Ratio estimates for PFOA, PFHxS and PFDA (Table 3a,b and Fig. 1) were not critically different from ORs for PFOS and PFOA, although statistical significance was not reached according to 95% Confidence Intervals. These similarities make the use of mixture-approaches highly important in future studies.

A PFOS level in the fourth quartile of exposure was, as compared with first quartile, related to an odds ratio for preeclampsia of the same size as for nulliparity, which is a well-established and major risk factor^5^. Hence, we find that from a clinical point of view, the present findings are of public health importance, especially having in mind that preeclampsia is common and the well- known serious health consequences to both women and offspring.

## Conclusions

A doubling of early pregnancy serum concentrations of perfluoroalkyl substances (PFOS and PFNA) were significantly associated with increased odds ratios for preeclampsia in the range of 1.38–2.02 according to adjusted analyses. The present associations between PFOS exposure and preeclampsia are in line with the limited previous research in higher exposed populations, but was here shown at a comparably lower exposure level. Overall, the associations consistent with established relations between prenatal PFAS exposures and birth weight may indicate toxicological pathways related to inflammation and oxidative stress. Since preeclampsia pose serious consequences to pregnant women and their fetuses, the here found risk estimates must be considered clinically relevant. Our findings therefore warrants urgent further studies, and if replicated, they support preventive efforts to further reduce community exposure.

## Methods

SELMA is a longitudinal pregnancy cohort study designed to investigate early life exposure to chemical compounds in the environment and the relation to growth, development and chronic diseases in children. The study recruited 2,582 pregnant women in the county of Värmland, Sweden, between September 2007 and March 2010^33^.

### Chemical analysis of PFAS in serum

Blood serum samples were obtained from 2,355 pregnant women in median week 10, (where 96.1% of the samples were collected before week 13) at their first visit at their antenatal care centre in Värmland, Sweden^33^.

Serum was analysed by using liquid chromatography-tandem-mass-spectrometry (LC/MS/MS) at The Department of Occupational and Environmental Medicine in Lund, Sweden. Eight PFAS were analysed including PFOS, PFOA, perfluorohexane sulfonate (PFHxS), perfluorononanoic acid (PFNA), perfluorodecanoic acid (PFDA), perfluoroundecanoic acid (PFUnDA), perfluoroheptanoic acid (PFHpA) and perfluorododecanoic acid (PFDoDA). A detailed description of the method is presented in Lindh, *et al*.^34^. Briefly, aliquots of 100 μl serum were added to 25 μl of a water:acetonitrile (50:50) solution containing labelled internal standards for all measured compounds. Proteins were precipitated by acetonitrile and vigorously shaking for 30 minutes. The samples were then centrifuged and the supernatant analysed using a LC (UFLCXR, SHIMADZU Corporation, Kyoto, Japan) connected to a hybrid triple quadrupole linear ion trap mass spectrometer (QTRAP 5500, AB Sciex, Foster City, CA, USA). Compounds with <50% of samples above LOD (Table 2) were excluded from analyses in relation to preeclampsia, where all values below LOD were set to half the LOD. The laboratory is part of a quality control program between analytical laboratories for analyses of PFOS and PFOA, coordinated by Professor Hans Drexler, Institute and Outpatient Clinic for Occupational, Social and Environmental Medicine, University of Erlangen-Nuremberg, Germany.

### Analyses of cotinine in serum as a marker of smoking

As described in Lindh, *et al*.^34^ prenatal serum samples were also analysed by LC/MS/MS for cotinine as a marker for smoking. The LOD for cotinine was 0.2 ng/mL. If cotinine levels were above LOD, subjects were categorized as smoke exposed (i.e either active or passive smokers).

### Obstetric diagnoses from the national birth register

In Sweden, antenatal care is free for all women. Normally women visit antenatal clinics from gestational week 8–12 and then regularly during pregnancy. Blood pressure and urinary albumin are routinely measured at each visit. Preeclampsia is diagnosed according to the *International Classification of Diseases, 10*^*th*^ *Revision*, based on systolic blood pressure ≥140 mmHg and/or diastolic blood pressure ≥90 mmHg accompanied by proteinuria after 20 pregnancy weeks. The Swedish Medical Birth Register includes virtually all deliveries in Sweden, and all maternal antenatal and perinatal obstetric diagnoses are recorded after the delivery. From the register we collected data on maternal diagnoses for identification of preeclampsia cases within the cohort, and co-variates.

### Statistical analysis

Maternal serum PFAS concentrations were transformed with the base-2 logarithm for reaching normal distribution. Univariate analyses of associations between potential confounders and preeclampsia were assessed with Chi^2^, Fisher’s Exact and-Whitney U-test (depending on data properties) and expressed with a p-value.

Logistic regression models were used to estimate odds ratios (ORs) for preeclampsia with increasing PFAS levels. The different PFAS were assessed compound by compound and ORs were calculated per unit increase in log base-2 PFAS concentrations (i.e., one unit in the log base-2 scale is a doubling of exposure), and also per quartiles of exposure with the first quartile as reference. Potential confounders included as co-variates were parity (nulliparous vs. parous), maternal body weight at enrolment to the study (kg), mothers age (years) and smoke exposure for the mother (yes vs no) according to cotinine in serum. Analyses were also performed after exclusion of multiple pregnancies. Since there were no cases of chronic hypertension among our preeclampsia cases, and only one woman with pre-pregnancy diabetes mellitus, we did not adjust for these established preeclampsia risk factors. No data on family history or preeclampsia during previous pregnancies were available. We found no reason to expect seasonal variations in exposure levels that together with the known seasonal pattern in preeclampsia occurrence may confound the analyses. This was however also confirmed in uni-variate analyses, and we performed post-hoc modelling where sampling calendar month and education level (university degree vs lower) were included among the regression model co-variates. Since levels of PFAS in women have been shown to decrease after a recent delivery^14^, we performed *a priori* the same regression analyses as above, restricted to women nulliparous at recruitment.

Statistical analyses were made using IBM SPSS Statistics for Windows, Version 22.0. (NY: IBM Corp, USA).

The study was performed in accordance with the Declaration of Helsinki and the Regional Ethical Review Board in Uppsala, Sweden approved the study protocol. Written informed consent was obtained from each participating woman.

## Supplementary information

Table 4 and Figures 2–4

## Data Availability

Data on chemical exposures (PFAS and cotinine) can be made available to researchers upon request (subject to a review of secrecy). Requests for data should be made to the Head of Deptartment of Health Sciences, Karlstad University. However, according to the Ethical Review Board decision and obtained personal consent, clinical data cannot be made freely available. This since as they are subject to secrecy in accordance with the Swedish Public Access to Information and Secrecy Act [OSL 2009:400]. Unique combinations of such data will make a study participant (i.e patient) identifiable, and consequently no clinical data will be shared.
