# Supplementary material for: Early pregnancy serum levels of perfluoroalkyl substances and risk of preeclampsia in Swedish women
Source: Sci Rep. 2019 Jun 24;9:9179. doi: 10.1038/s41598-019-45483-7 (PMC6591359; doi:10.1038/s41598-019-45483-7)
Supplement: Supplementary file 1 — Table 4 and Figures 2–4 [file 41598_2019_45483_MOESM1_ESM.docx]

**Early pregnancy serum levels of perfluoroalkyl substances and risk of preeclampsia in Swedish women**

Sverre Wikström, Christian H. Lindh, Huan Shu, Carl-Gustaf Bornehag

**Table 4.** Bi-variate correlations (r) between log base(2)-transformed serum concentrations (ng/mL) of respectively PFAS, sampled at median 10 weeks of pregnancy in 1,773 women. All correlations were significant at p<0.001 level.

| **Compound** | PFOS | PFOA | PFHxS | PFNA | PFDA | PFUnDA | PFHpA |
| --- | --- | --- | --- | --- | --- | --- | --- |
| PFOS |  | 0.60 | 0.36 | 0.55 | 0.57 | 0.47 | 0.13 |
| PFOA | 0.60 |  | 0.29 | 0.72 | 0.66 | 0.32 | 0.42 |
| PFHxS | 0.36 | 0.29 |  | 0.18 | 0.21 | 0.16 | 0.08 |
| PFNA | 0.55 | 0.72 | 0.18 |  | 0.76 | 0.53 | 0.32 |
| PFDA | 0.57 | 0.66 | 0.21 | 0.76 |  | 0.73 | 0.32 |
| PFUnDA | 0.47 | 0.32 | 0.16 | 0.53 | 0.73 |  | 0.16 |
| PFHpA | 0.13 | 0.42 | 0.08 | 0.32 | 0.32 | 0.16 |  |

**Figure 2.**


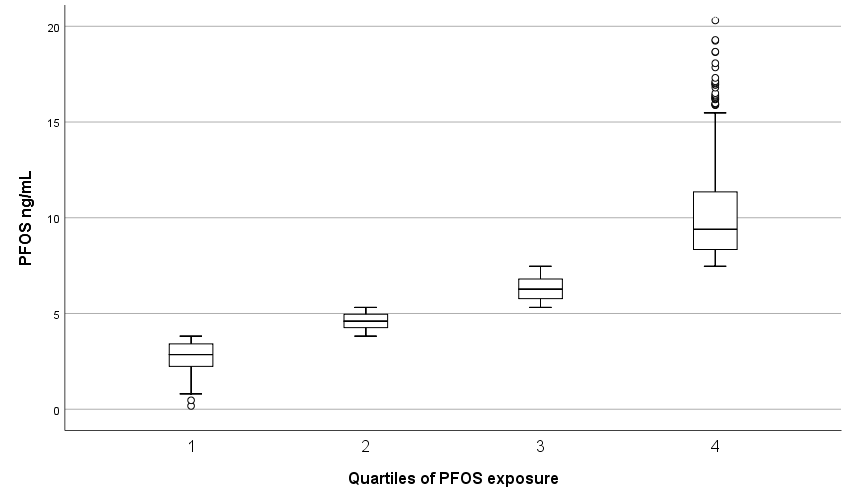


**Figure 3.**


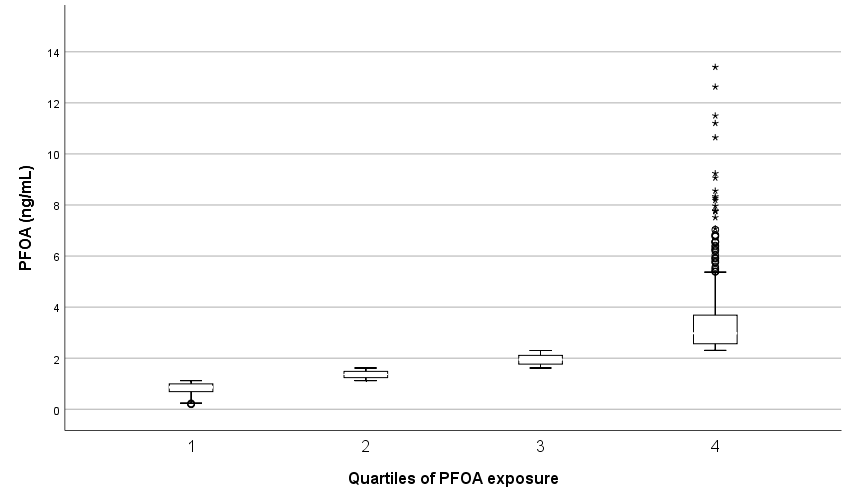


**Figure 4.**


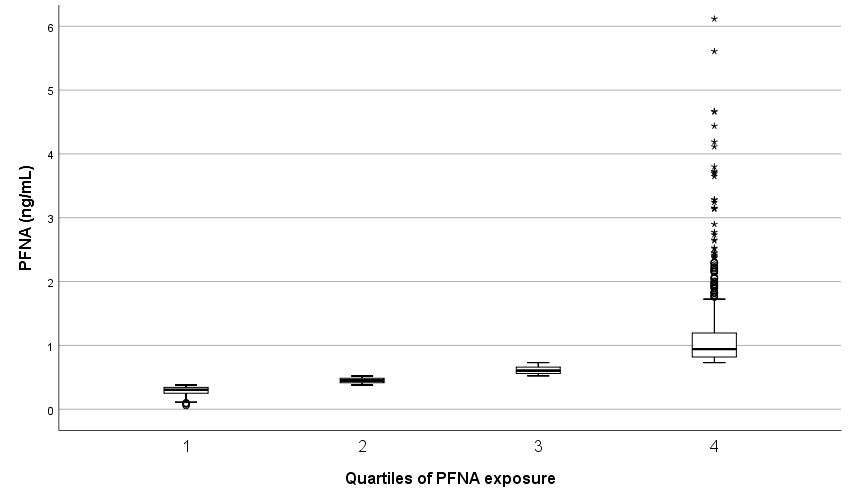


**Figures 2-4.** PFOS, PFOA and PFNA concentrations (ng/mL) displayed per quartile of exposure. Boxes representing 25^th^ to 75^th^ percentile within each quartile group.
